# Accuracy of the FY-L formula in calculating intraocular lens power after small-incision lenticule extraction

**DOI:** 10.3389/fmed.2023.1241824

**Published:** 2023-08-24

**Authors:** Yingfeng Hu, Liqun Lin, Danqi Zeng, Yan Wang, Rong Zhang, Zhe Zhang, Zheng Wang, Guangbin Zhang, Xiangyu Ye

**Affiliations:** ^1^Fuzhou Eye Hospital, Fuzhou, Fujian, China; ^2^Xianyou County General Hospital, Putian, Fujian, China; ^3^Xiamen Eye Center, Xiamen University, Xiamen, Fujian, China; ^4^Shenzhen Eye Hospital, Jinan University, Shenzhen, Guangdong, China; ^5^Shenzhen Eye Institute, Shenzhen, Guangdong, China

**Keywords:** intraocular lens power calculation, FY-L formula, accuracy, theoretical model, small-incision lenticule extraction

## Abstract

**Purpose:**

The study aimed to assess the accuracy of the FY-L formula in calculating intraocular lens (IOL) power after small-incision lenticule extraction (SMILE).

**Methods:**

For the post-SMILE IOL calculation of the same eye, the IOL power targeting the pre-SMILE eyes' lowest myopic refractive error was used. The FY-L formula, the Emmetropia Verifying Optical Formula (EVO-L), the Barrett True-K no history, and the Shammas-L, respectively, were used to calculate the predicted refractive error of target IOL power. A comparison was made between the change in spherical equivalent induced by SMILE (SMILE-Dif) and the variance between IOL-Dif (IOL-Induced Refractive Error) before and after SMILE. The prediction error (PE) was defined as SMILE-Dif minus IOL-Dif. The proportion of eyes with PEs within ±0.25 D, ±0.50 D, ±0.75 D, and ±1.00 D, the numerical and absolute prediction errors (PEs and AEs), and the median absolute error (MedAE) were compared.

**Results:**

In total, 80 eyes from 42 patients who underwent SMILE were included in the study. The FY-L formula generated the sample's lowest mean PE (0.06 ± 0.76 D), MAE (0.58 ± 0.50 D), and MedAE (0.47 D), respectively. The PEs in ±0.25 D, ±0.50 D, ±0.75 D, and ±1.00 D comprised 28.8%, 46.3%, 70.0%, and 87.5%, respectively, for the FY-L formula. Compared to other formulas, the FY-L formula produced the highest value with PEs for the percentage of eyes in ±0.50 D, ±0.75 D, and ±1.00 D.

**Conclusion:**

This study demonstrates that the FY-L formula provides satisfactory outcomes in estimating the IOL power in the eyes after SMILE.

## Introduction

The number of laser refractive procedures has significantly increased during the last three decades. Small incision lenticule extraction (SMILE), one of the most well-known and established keratorefractive treatments for the correction of myopia, has been used in more than five million surgeries worldwide (1). However, it is difficult to attain the intraocular lens (IOL) estimation specifications for these people while they are having cataract surgery. The two main reasons that IOL power calculations after refractive surgery went wrong were an inaccurate measurement of keratometry (K) value and incorrect predictions of effective lens position (ELP) (2, 3). After the corneal refractive procedure, the cornea's anterior–posterior surface relationship is changed (4, 5). The predicted total corneal power based on the anterior corneal power could be erroneous when the standardized value (1.3375) for the refractive index of the cornea is applied (6, 7). In order to predict the ELP, several IOL calculation formulas use corneal power values. Incorrect corneal refractive power leads to incorrect ELP.

To overcome these limitations, numerous theoretical and empirical strategies have been developed. Various methods, including Feiz/Mannis (7), clinical history (8), and the corneal bypass method (9), employ preoperative k and changes in manifest refraction to estimate corneal power. Aramberri (3) suggested using the double-K method to prevent the ELP-related IOL prediction error. Various methods that do not need preoperative information have been developed, including Barrett True-K no history, Shammas-L, EVO-L, and the American Society of Cataract and Refractive Surgery (ASCRS) average method (2, 10, 11). Some researchers found that the Barrett True-K appears to have a greater accuracy among these methods in eyes after myopic laser *in situ* keratomileusis (LASIK) or photorefractive keratectomy (PRK) (12–14). A new no-history method named FY-L was developed for eyes that underwent LASIK or PRK, which utilizes the corrected keratometric value combined with an optimized ELP prediction method. In this study, the accuracy of various IOL power calculation formulas, including FY-L, Barrett True-K no history with and without the posterior corneal power, Emmetropia Verifying Optical Formula (EVO-L), and Shammas-L, was assessed in eyes with SMILE.

## Methods

### Patients and surgery

This prospective study was consented to by the Fuzhou Eye Hospital (FZYKYY-KY-2023-007) and conformed to the Declaration of Helsinki. All patients provided written consent. In total, 80 eyes from 42 patients who underwent SMILE between September 2022 and December 2023 in Fuzhou Eye Hospital and Xiamen Eye Center Affiliated with Xiamen University were included in the study. The VisuMax femtosecond laser (Carl Zeiss Meditec AG) was used for the SMILE treatments, which were performed by experienced refractive surgeons.

The eligibility requirements for enrollment included refraction stability for 2 years, 20/20 corrected distance visual acuity, and successful SMILE surgeries with no complications that occurred during or after surgery. Exclusion criteria included corneal disease, severe dry eye, serious intraoperative or postoperative complications that would distort data on the final refractive outcome, ocular infection, any optical opacities or pathology shown after slit-lamp examination, prior intraocular surgery, corneal surgery, or ocular trauma.

### Preoperative and postoperative measurements

Every patient underwent a complete ophthalmic examination before and after SMILE, including uncorrected distance visual acuity, slit-lamp examination, tonometry, fundoscopy, corrected distance visual acuity, and manifest and cycloplegic refraction. IOLMaster 700 (Carl Zeiss Jena, Germany) was employed to measure the following measurements: axial length (AL), anterior chamber depth (ACD), keratometry (K), lens thickness (LT), and white to white (WTW). Corneal topography and posterior corneal power were examined using the Pentacam (Oculus, Wetzlar, Germany). At least 3 months after the operation was completed, the postoperative examination was performed.

### IOL power calculation

To emulate cataract surgery in post-SMILE eyes, a theoretical calculation model was presented. It is assumed that all patients are implanted with monofocal IOL (SN60WF, Alcon Laboratories, Inc.) with the following constants: for Haigis, a0 = 1.839, a1 = 0.4, and a2 = 0.1, and 118.9 for the other formulas. The Haigis formula was used for calculating the pre-SMILE IOL power. The post-SMILE IOL power was calculated using four methods, including the FY-L formula, the Barrett True-K no history formula, the EVO-L formula, and the Shammas-L formula, respectively. The Barrett True-K no history formula was calculated in two modes, including using the predicted corneal power of the posterior surface (PPCP) and the actual measured corneal power of the posterior surface (MPCP). For the post-SMILE IOL calculation of the same eye, the IOL power targeting the pre-SMILE eyes' lowest myopic refractive error was used.

The FY-L formula used the Actual Ka + p method to modify the measured corneal power (15). The corneal power was calculated as follows: K_corrected_ = 1.114 × (K_flattest_ + K_steepest_)/2 + K_posterior_, where K_flattest_ and K_steepest_ were measured by the IOL master 700, and K_posterior_ was measured by Pentacam.

To predict the ELP, the FY-L formula was based on the FY-IOL formula, which uses the ACD, anterior and posterior corneal power, LT, and AL for calculation.

### Calculation of the prediction error

The evaluation was performed on the differences in the IOL-induced refractive error (IOL-Dif) before and after SMILE (16). For example, A +12.00 diopter (D)-IOL was selected to target postoperative refraction of −0.20 D before SMILE. After SMILE, the same IOL was used to calculate the resulting target refraction (4.50 D). The IOL difference (IOL-Dif) would be 4.50 D- (−0.20 D) = 4.70 D. The difference caused by the SMILE surgery in preoperative and postoperative manifest subjective refraction is defined as SMILE-Dif. IOL-Dif minus SMILE-Dif was used to define the prediction error (PE). Negative values show a myopic shift and positive values show a hyperopic shift. The proportion of eyes with PEs within ±0.25 D, ±0.50 D, ±0.75 D, and ±1.00 D, the numerical and absolute prediction errors (PEs and AEs), and the median absolute error (MedAE) were calculated for each formula to assess the predictive accuracy. In this study, we did not adjust the mean PE to zero for two reasons: (1) All patients were simulated with the same IOL implanted, and an optimized IOL constant was used for IOL calculations and (2) in the normal clinical scenario, the post-refractive patient has a relatively large range of refractive error, and optimization of IOL constants may not be very effective. The surgeons routinely use the optimized IOL constants from patients with normal cataracts and do not have specific optimized lens constants for eyes after refractive surgery for IOL calculations.

### Statistics

Statistical analysis was performed using SPSS software (version 22.0, SPSS Inc., Chicago, USA). The normality of the data was checked using the Kolmogorov–Smirnov test. A generalized estimating equation (GEE) procedure was used to adjust the correlation between the right and left eyes. Differences in PEs and AEs for all methods were compared by a GEE model and included Bonferroni multiple comparisons. The Cochran *Q*-test was used to compare the percentage of eyes with a PE within each interval. Any statistically significant difference found in Cochran's *Q* was determined using McNemar's test with Bonferroni adjustment. A *P*-value of < 0.05 was considered to be statistically significant.

## Results

**Figure 1:**
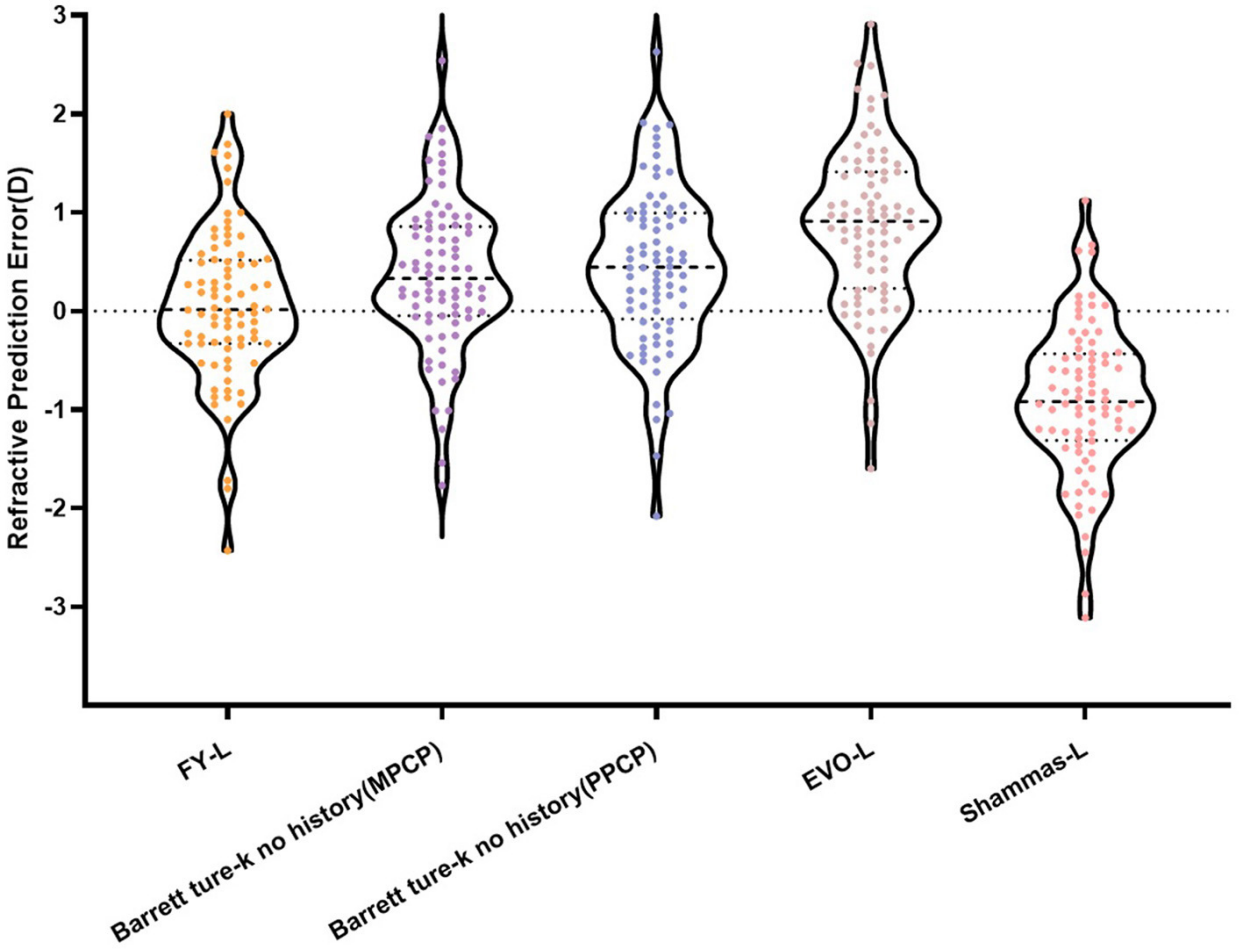
Distribution and quartile ranges of the PEs.

**Figure 2:**
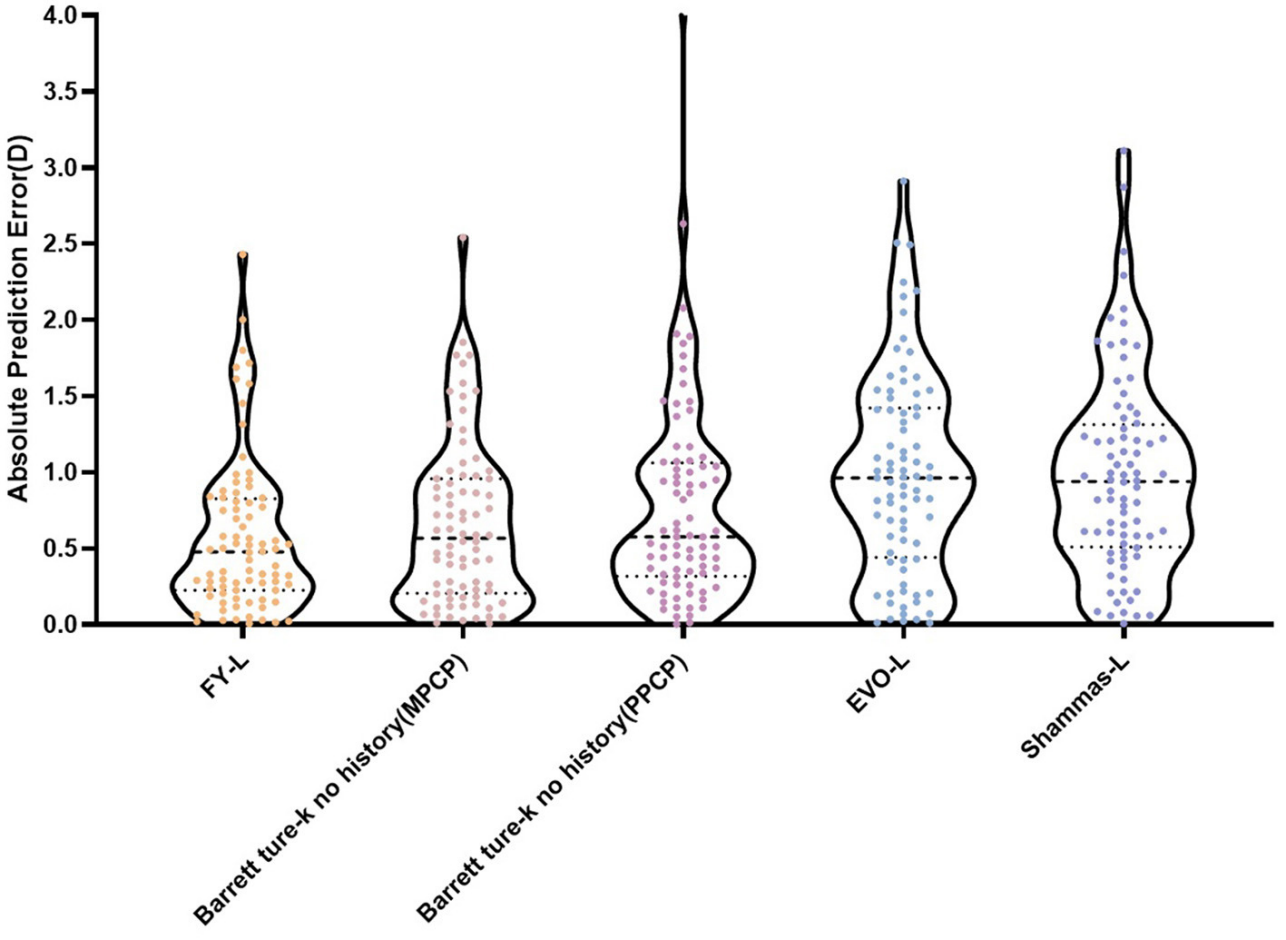
Distribution and quartile ranges of the AEs.

Table 1 sums up all the biometrics and demographics of the study population. The study comprised 80 eyes of 42 patients. The study contained 24 men and 18 women. The mean age was 23.54 ± 4.03 years. The mean SE before SMILE was −4.95 ± 1.66 D. After SMILE, the mean SE was 0.04 ± 0.58 D. The mean SMILE-Dif was 4.87 ± 1.95 D. Figures 1, 2 show the distribution and quartile ranges of the PEs and the AEs for each formula, respectively. Table 2 summarizes the mean PE, MedAE, MAE, and the percentages with PEs within ±0.25 D, ±0.50 D, ±0.75 D, and ±1.00 D in the 80 eyes retrospectively, while various formulas were used.

**Table 1:** Data sample characteristics (*n* = 80).

**Parameter**	**Measurement before SMILE**	**Measurement after SMILE**	***P*-value**
**Sex (F/M)**			
*n*		18/24	
(%)		42.9%/57.1%	
**Eye (OD/OS)**			
*n*		40/40	
(%)		50%/50%	
**Age (y)**			
Mean ± SD		23.54 ± 4.03	
Range		17, 33	
**Manifest refraction spherical equivalent (D)**			
Mean ± SD	−4.95 ± 1.66	0.04 ± 0.58	< 0.001
Range	−9, −1.5	−2.5, 1.25	
**Axial length (mm)**			
Mean ± SD	25.50 ± 1.06	25.37 ± 1.05	< 0.001
Range	23.28, 28.11	23.20, 27.84	
**Anterior chamber depth (mm)**			
Mean ± SD	3.72 ± 0.26	3.53 ± 0.27	< 0.001
Range	3.18, 4.33	3.18, 4.33	
**Lens thickness (mm)**			
Mean ± SD	3.57 ± 0.21	3.65 ± 0.22	< 0.001
Range	3.15, 4.01	2.98, 4.17	
**Mean keratometry (D)**			
Mean ± SD	43.79 ± 1.29	39.30 ± 1.29	< 0.001
Range	40.84, 46.41	35.87, 44.11	
**Mean posterior keratometry (D)**			
Mean ± SD	−6.34 ± 0.20	−6.33 ± 0.21	0.083
Range	−6.7, −5.90	−6.7, −5.80	

F, female; M, male; IOL, intraocular lens; mm, millimeters; D, diopters; OD, right eye; OS, left eyes; SMILE, small-incision lenticule extraction.

**Table 2:** Mean prediction error, mean absolute prediction error, median of absolute prediction error, and percentage within a specific refractive error range.

**IOL formula**	**PE (D)**	**MAE (D)**	**MedAE (D)**	**±0.25 D (%)**	**±0.50 D (%)**	**±0.75 D (%)**	**±1 D (%)**
FY-L	0.06 ± 0.76	0.58 ± 0.50	0.47	28.8%	46.3%	70.0%	87.5%
Barrett True-k no history (MPCP)	0.36 ± 0.76	0.64 ± 0.51	0.55	30.0%	46.3%	62.5%	78.8%
Barrett True-k no history (PPCP)	0.47 ± 0.90	0.74 ± 0.62	0.58	20.0%	42.5%	58.8%	70.0%
EVO-L	0.85 ± 0.82	0.93 ± 0.64	0.92	18.8%	26.3%	36.3%	53.8%
Shammas-L	−0.91 ± 0.77	1.04 ± 0.67	0.98	13.8%	23.8%	40.0%	58.8%
*P*-value	< 0.001^a^	< 0.001^b^	n.s.	0.011^c^	< 0.001^c^	< 0.001^c^	< 0.001^c^

FY-L, FY-L formula; Barrett True-k no history (PPCP), Barrett True-K no history formula without posterior corneal power; Barrett True-k no history (MPCP), Barrett True-K no history formula with posterior corneal power; EVO-L, EVO-L formula; Shammas-L, Shammas-L formula; D, diopters; MAE, mean absolute prediction error; MedAE, median absolute prediction error; PE, mean numerical prediction error.

a Difference among the PEs.

b Difference among the MAEs.

c Difference among the percentages of eyes within each PE.

The FY-L formula generated the sample's lowest PE (0.06 ± 0.76 D) and MedAE (0.47D). The general difference between the PEs had statistical significance (*P* < 0.001), and the *post-hoc* analysis showed a statistical difference between all comparisons, with *P*-values of < 0.001, except *P* = 0.041 in Barrett True-K no history (MPCP) vs. the Barrett True-K no history (PPCP).

The average values of the AEs employing the FY-L formula were 0.58 ± 0.50 D (range, 0.01–2.43 D), 0.64 ± 0.51 D (range, 0.01–2.54 D) for the Barrett True-K no history (MPCP), 0.74 ± 0.62 D (range, 0.00–4.12 D) for the Barrett True-K no history (PPCP), 0.93 ± 0.64 D (range, 0.01–2.91 D) for the EVO-L formula, and 1.04 ± 0.67 D (range, 0.00–3.13 D) for Shammas-L formula. The GEE procedure found differences that were statistically significant between the AEs (*P* < 0.001). The posttest found statistical differences between FY-L vs. EVO-L and FY-L vs. Shammas-L, with *P*-values of < 0.001 and 0.001, respectively. The comparison of Barrett True-K no history (MPCP) with EVO-L and Shammas-L also showed statistical differences with *P*-values of < 0.001 and 0.013. Other statistically significant comparisons were between Barrett True-K no history (PPCP) vs. EVO-L and EVO-L vs. Shammas-L, with *P-*values of 0.001 and < 0.001. No differences were found in the rest of the comparisons.

**Figure 3:**
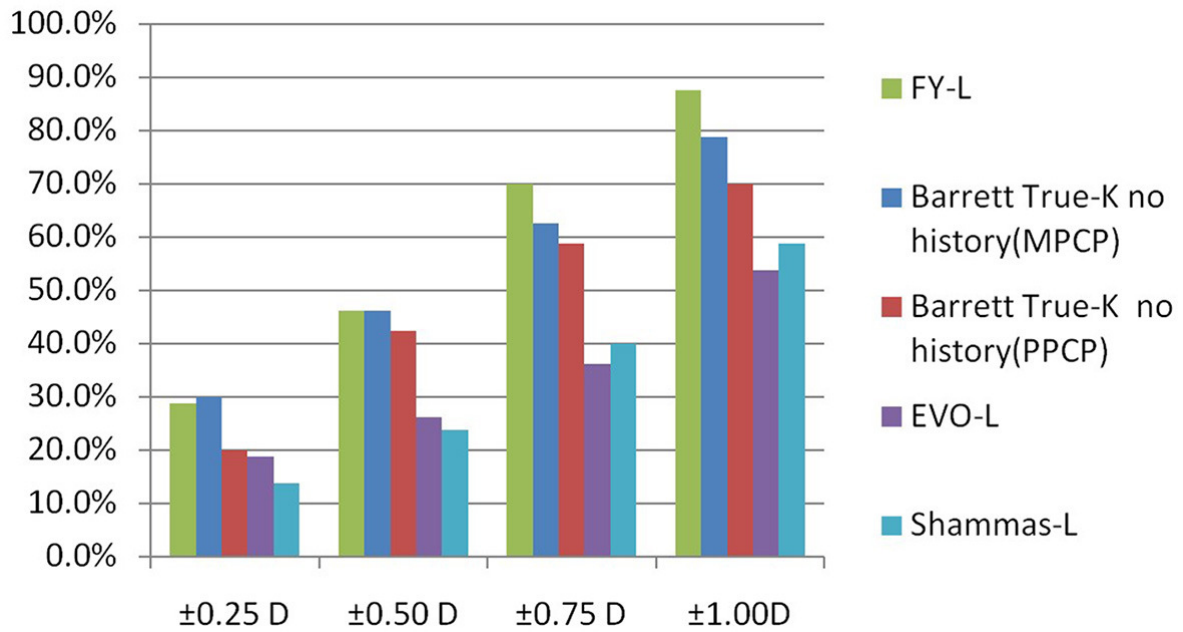
Evaluation of the proportion of eyes within specific ranges of prediction errors.

The prediction errors within ±0.25 D were 28.8%, 46.3% within ±0.50 D, 70% within ±0.75 D, and 87.5% within ±1.00 D, respectively, for the FY-L formula. Compared the FY-L formula with other formulas, it produced the highest value for the proportion of PEs with ranges between ±0.50 D and ±0.75 D as well as ±1.00 D (Figure 3). The following comparisons of proportions within ±0.25 D found statistical differences: FY-L vs. Shammas-L (*P* = 0.034), Barrett True-K no history (MPCP) vs. Barrett True-K no history (PPCP; *P* = 0.011), and Barrett True-K no history (MPCP) vs. Shammas-L (*P* = 0.028). Concerning the proportion of ±0.5 D, the comparison of FY-L with Barrett True-K no history (PPCP), FY-L with EVO-L, and FY-L with Shammas-L showed statistical differences with *P*-values of 0.033, < 0.001, and < 0.001. There were statistically significant differences between Barrett True-K no history (MPCP) and EVO-L vs. Barrett True-K no history (MPCP) vs. Shammas-L, with both *P*-values of 0.001 for the comparison. Other statistically significant comparisons were between Barrett True-K no history (PPCP) vs. EVO-L and Barrett True-K no history (PPCP) vs. Shammas-L with *P*-values of 0.001 and 0.006. Concerning the proportion between ±0.75 D, the comparison of FY-L with EVO-L and FY-L with Shammas-L showed statistical differences with both *P*-values of < 0.001. The comparison of Barrett True-K no history (MPCP) with EVO-L, Shammas-L, and Barrett True-K no history (PPCP) showed statistical differences with both *P*-values of < 0.001. The comparison of Barrett True-K no history (PPCP) with EVO-L and Shammas-L also showed statistical differences with *P*-values of < 0.001 and 0.002. Concerning the proportion of ±1.0 D, the comparison of FY-L vs. EVO-L, FY-L vs. Shammas-L, and FY-L vs. Barrett True-K no history (PPCP) were significantly different with both *P*-values of < 0.001. The comparison of Barrett True-K no history (MPCP) with EVO-L, Shammas-L, and Barrett True-K no history (PPCP) showed statistical differences with *P*-values of < 0.001, < 0.001, and 0.021. The comparison of Barrett True-K no history (PPCP) with EVO-L and Shammas-L also showed statistical differences with *P*-values of < 0.001 and 0.015. Between the remaining groups, there were no statistically significant differences.

## Discussion

Due to its safety, effectiveness, predictability, and lack of issues connected to valves, small-incision lenticule extraction (SMILE) is preferred among patients (17, 18). Given an aging population, the number of patients who have been previously treated with SMILE and require cataract surgery is predicted to increase. It is achievable to precisely calculate IOL power after PRK or LASIK using a variety of empirically optimized formulas that are available as online calculators, with or without preoperative refractive data (19). In the methods that do not demand clinical history information of the patients, the FY-L formula used the actual Ka + p method to adjust the measured corneal power, which was based on our previously published study in 2010 (15). The Adult Eye Preferred Practice Pattern^®^ guidelines (PPP), which were written by members of the Cataract and Anterior Segment Preferred Practice Pattern^®^ Panel, mentioned this method in 2017 and 2021 (20, 21). To estimate the ELP, the FY-L formula was based on the FY-IOL formula, which was optimized in groups based on different AL, ACD, anterior and posterior corneal power, and LT. In prior studies, the FY-L formula displayed high precision in IOL power predictions post-LASIK or PRK (15, 22). In our study, the FY-L formula was compared to three established IOL power calculation formulas, including EVO-L, Shammas-L, and Barrett True-K no history, in eyes receiving cataract surgery after SMILE. The same IOL was virtually implanted before and after SMILE in the theoretical model of this study.

It is considered that the IOL-Dif should be equivalent to the SMILE-Dif (23, 24). The IOL power was calculated employing the Haigis formula for the following there reasons before SMILE: (1) Since this study is based on a theoretical model and not on actual IOL implantation, the postoperative refractive outcomes are simulated data. Using the same formula before and after surgery may lead to errors in the calculations. Barrett True-K no history was based on Barrett Universal II for the IOL power calculations. We intend to use a formula before SMILE that does not correlate with the formulas used for the IOL power calculations after SMILE. (2) The patients in this study had an AL range between 23.28 and 28.11 mm, and both clinical application and previous studies showed the good accuracy of the Haigis formula for IOL calculations in this population (25–27). (3) The Haigis formula has been published publicly for its calculation; therefore, it is easier to optimize the IOL constants with this formula.

In the present study, calculations with the FY-L formula yielded very accurate results with an average PE 0.06 ± 0.76 D, an average AE 0.58 D, and a MedAE 0.47 D. Thereby, 87.5% of the eyes analyzed fell within the range of ±1 D, while 70% were within ±0.75 D, and 46.3 and 28.8% of the eyes were within the limits of ±0.50 and ±0.25 D, respectively. Our investigation found promising outcomes with the Barrett True-K no history for cases with actual posterior corneal power, with an average PE of 0.36 ± 0.76 D, an average AE of 0.64 D, and an MAE of 0.55 D. In total, 78.8% of the eyes examined were within ±1 D, 62.5% were within ±0.75 D, 46.3% were in the ±0.5 D range, and 30.0% were in the ±0.25 D range for Barrett True-K no history (MPCP). The FY-L formula shows similar predictability as the Barrett True-K formula when considering the measured cornea power of the posterior surface. The accuracy of the Barrett True-K no history formula was slightly reduced when the corneal power of the posterior surface was not imputed but estimated. According to Savini et al., the Barrett True-K formula had better accuracy with the input of examination of the corneal posterior surface and preoperative details of the keratorefractive operation (28). In prior studies, corneal powers substantially altered in the anterior corneal surface while not changing in the posterior corneal surface, both LASIK, PRK, and SMILE (29). Our data suggest the same change. Therefore, the post-SMILE IOL power estimate would be more reliable if the formulas used both anterior and posterior corneal power. In Meziane Elotmani's study, calculations after SMILE with Okulix provided highly accurate results with an average PE of 0.002 ± 0.453 D, and 79% of the cases examined were within ±0.50D (24). However, the pre-SMILE IOL power was estimated employing multiple formulas rather than a common formula as a standard in the study. In addition, no other formulas that used measurements of both the anterior and posterior cornea, such as the Barrett True-K formula, were compared in the article. Lischke et al. also showed in research that ray tracing gives the greatest accuracy IOL power estimation after SMILE, with an average PE of 0.18 ± 0.48 D (30). The FY-L formula showed comparable results with these two formulas.

In our calculations of eyes after SMILE, the EVO-L formula yielded a tendency for a hyperopic shift, and the Shammas-L formula yielded a tendency for a myopic shift. The mean arithmetic PE of the EVO-L formula was 0.85 ± 0.82 D, and the MAE was 0.93 D. In this study, Shammas-L provided the lowest accuracy, with an average PE of −0.91 ± 0.77 D and the lowest ±0.50 D accuracy of 23.8% and ±0.25 D accuracy of 13.8%. Lischke et al. also showed in their research that the biggest IOL power overestimation was produced by the Shammas-L formula, with obtained a PE of −0.96 ± 1.14 D (30).

In summary, the outcomes of this study show that the FY-L formula offers satisfactory outcomes in estimating the IOL power in the eyes after SMILE. To the best of our knowledge, this is the first instance in which the FY-L formula has been used to determine the intraocular lens power after SMILE. In addition, the Barrett True-K no history formula increased its accuracy when the posterior corneal power was used instead of only the anterior corneal measurements. These outcomes show that, in eyes with prior SMILE, selecting the correct IOL provides significant benefits when the corneal posterior surface has been taken into consideration in IOL calculations.

This study had at least three limitations. First, a theoretical calculation model was presented in the study. The most accurate way to contrast multiple IOL power calculation methods would be by investigating the real postoperative refraction outcomes of cataract surgery after SMILE. However, these clinical data are currently unavailable. Second, the IOLMaster 700 and Pentacam HR, the equipment used in this study, apply different measurement techniques and might deliver different results. Therefore, in the follow-up study, the results of the calculation using the posterior corneal power obtained from different measurement devices should be compared and examined. Third, including the Ray Tracing Formula, Okulix, and Total Keratometry Method, which also used both anterior and posterior corneal measurements. In this article, there is no comparison of the methods mentioned above. In a follow-up study, we will compare these methods for a more comprehensive result.

## Data availability statement

The raw data supporting the conclusions of this article will be made available by the authors, without undue reservation.

## Ethics statement

The studies involving humans were approved by Medical Ethics Committee, Fuzhou Eye Hospital. The studies were conducted in accordance with the local legislation and institutional requirements. The participants provided their written informed consent to participate in this study.

## Author contributions

XY and GZ contributed to the concept and design of the study. YH and DZ performed the acquisition of data. YH and LL analyzed and interpreted the data and performed the statistical analysis. YH drafted the manuscript. LL, YW, and RZ performed a critical revision of the manuscript for important intellectual content. ZW and XY provide administrative, technical, or material support. All authors contributed to the article and approved the submitted version.
